# Evaluation of a Custom SNP Panel for Identifying and Rectifying of Misjudged Paternity in Deficiency Cases

**DOI:** 10.3389/fgene.2021.602429

**Published:** 2021-02-22

**Authors:** Liao Chang, Huiyun Yu, Xinyao Miao, Siqi Wen, Bao Zhang, Shengbin Li

**Affiliations:** ^1^Bio-evidence Sciences Academy, Western China Science and Technology Innovation Harbour, Xi’an Jiaotong University, Xi’an, China; ^2^College of Medicine and Forensics, Xi’an Jiaotong University, Xi’an, China; ^3^Forensic Genomics International, Beijing Genomics Institute-Shenzhen, Shenzhen, China

**Keywords:** single nucleotide polymorphism, misjudged paternity, random false match, ambiguous STR result, deficiency case, close relative

## Abstract

Parentage testing is routinely performed by genotyping short tandem repeat (STR) through capillary electrophoresis in the present. However, ambiguous or even misjudged paternity based on STRs happens from time to time in cases where only one putative parent is available. We analyzed STR data of 7,818,969 unrelated pairs and 75 close-relative pairs and found that although the probability of a random false match between non-relatives was 4.22 × 10^–6^, the incidence of false or ambiguous paternity results between children and first-degree relatives of their true parent was as high as 18.67%. These results highlight the risk of false inclusion of a relative or even non-relatives in parentage testing with STRs. We then validated all ambiguous STR results by targeted sequencing with a custom panel containing 4,830 individual identification single nucleotide polymorphisms (IISNP), found that the ratio of mismatch loci to total SNPs was 1.78–6.95% in close relatives compared with 10.93–13.49% in unrelated pairs. Last, we reported three real cases with undetermined paternity by STRs and rectified them by dissecting with our IISNP panel. These results suggested that high-density IISNP panel can be used to identify and rectify misjudged cases effectively.

## Introduction

Parentage testing plays a critical role in searching for missing persons (Yu and Fung, 2018), identifying disaster victims (Wright et al., 2018), solving inheritance disputes (Patidar et al., 2015), and immigration casework (Wenk and Shao, 2014). Presently, analysis of short tandem repeat (STR) markers through capillary electrophoresis (CE) is routinely used for parentage testing because of its high discrimination power (Butler et al., 2004).

Nevertheless, there are situations where the result based on the CE-STR method are ambiguous, mainly manifested by opposite conclusions drawn from different STR kits or by insufficient combined paternity index (CPI) value accompanied by separate genetic incompatibilities (Phillips et al., 2008). Ambiguous STR results are particularly common in cases where only one putative parent is available (also known as deficiency cases) (Borsting and Morling, 2011) or the alleged parent is a close relative to the real parent (Pinto et al., 2013), because it is difficult to distinguish the germline mutation from a real mismatch in the genetic inconsistency (Lindner et al., 2014). Using more STR loci could increase the confidence and thus alleviate the situation; however, current CE methods have limitation in multiplex capability due to the maximum of six dye channels. Moreover, STRs display mutation rate as high as 10^–3^–10^–4^ per generation (Legendre et al., 2007), so it is possible to observe more genetic incompatible loci in true parent–child pairs when adding STRs.

In addition, compared with the ambiguous STR results between close relatives, a random but perfect false match (RPFM) between unrelated individuals may also result in error. In other words, an unrelated person shares at least one allele with the child at all STR loci by chance and thus could be misjudged as the biological parent. RPFM may occur when searching for missing persons in a large population, such as finding the parents of rescued trafficked children in a large database (Yu and Fung, 2018).

Single nucleotide polymorphisms (SNPs) are complementary markers to STRs, with their feature of 10^4^–10^5^ times higher genetic stability (Nachman and Crowell, 2000) and greater abundance in the human genome than STRs (Auton et al., 2015). It has been reported that the discriminatory power of 50–60 SNPs with a minor allele frequency (MAF) larger than 0.3 is comparable to 15 STRs (Borsting et al., 2008). Consequently, the SNP*for*ID 52-plex SNP panel is often used to solve the question of ambiguous paternity based on STRs (Borsting et al., 2008; Borsting and Morling, 2011; Lindner et al., 2014). However, an ambiguous paternity based on STRs may result from mutational events or presence of null alleles in true parenthood and may also result from false matches between close relatives or even non-relatives. When we face an ambiguous result, how to determine the reason from scratch then rectify the conclusion efficiently remains unclear.

In this study, we first estimated the prevalence of ambiguous paternity in cases involving close relatives as well as the probability of random false match between non-relatives with STRs. Then we evaluated the performance of a custom panel with 4,830 individual identification SNPs (IISNP) in the above situations. Last, we reported three real cases with undetermined STR results and rectified them by dissecting with our IISNP panel.

## Materials and Methods

### Sample Collection

This study was approved by the Institutional Review Board Administration in Xi’an Jiaotong University (No. 2018-408). We collected three batches of samples: (1) 55 samples from eight families for close relative matching experiment, (2) 64 samples in 33 randomly false matched pairs, and (3) 6 samples in three cases with ambiguous STR results. For each participant, informed written consent was obtained, then blood sample was spotted on the anti-bacterial nucleic acid collection card (Nuhighbio, China).

### STR Genotyping

Blood genome DNA was extracted using the TIANamp Micro DNA kit (TIANGEN, China) according to the manufacturer’s protocol. Extracted DNA was quantified with Qubit^®^ dsDNA HS Assay Kits on Qubit^®^ Fluorometer 3.0 (Life Technologies, United States).

For close relative samples, 1 ng genome DNA was amplified using the HUMAN DNA Typing-YanHuang kit (FGI, China) on the thermal cycler (GeneAmp polymerase chain reaction 9700, Thermo Fisher Scientific, United States). Then, STR genotyping of the PCR products was performed on the ABI 3500 genetic analyzer and analyzed with GeneMapper ID-X software (Thermo Fisher Scientific, United States). HUMAN DNA Typing-YanHuang kit contains 24 autosomal STR loci (D22S1045, SE33, D2S441, D6S0143, D1S1656, D10S1248, CSF1PO, D12S391, D13S317, D16S539, D18S51, D19S433, D21S11, D2S1338, D3S1358, D5S818, D7S820, D8S1179, FGA, Penta D, Penta E, TH01, TPOX, vWA).

For randomly matched study, existing STR data (STRtyper-21G kit, HEALTH Gene Technologies, China) of 3,955 unrelated individuals from our DNA center were collected after approval. STRtyper-21G kit contains 20 autosomal STR loci (D6S1043, D1S1656, CSF1PO, D12S391, D13S317, D16S539, D18S51, D19S433, D21S11, D2S1338, D3S1358, D5S818, D7S820, D8S1179, FGA, Penta D, Penta E, TH01, TPOX, vWA). We note that all STR kits in this study are commonly used commercial products, which are often used for parentage testing alone or in combination.

### Probability of RPFM in Duos

According to the research from Ayres (2002) and Yu and Fung (2018), consider a locus *r* having *n* alleles *A*_1_, *A*_2_, …, *A*_*n*_ with corresponding allele frequency *p*_1_, *p*_2_, …, *p*_*n*_. The probability of excluding a random person from parentage at locus *r* is given by *Q*_*r*_(*θ*), where *θ* is the coancestry coefficient. *θ* = 0 implies the population is in Hardy–Weinberg equilibrium (HWE).

$$Q_{r}\,\left(\theta \right)=\frac{\left(1-\theta \right)}{\left(1+\theta \right)\,\left(1+2\,\theta \right)}[\sum_{i=1}^{n}p_{i}\left(\theta +\left(1-\theta \right)p_{i}\right)\left(1-p_{i}\right)\left(\theta +\left(1-\theta \right)\,\left(1-p_{i}\right)\right)+\sum_{i=1}^{n-1}\sum_{j=i+1}^{n}2\,p_{i}\,p_{j}\,\left(1-\theta \right)\left(1-p_{i}-p_{j}\right)\left(\theta +\left(1-\theta \right)\left(1-p_{i}-p_{j}\right)\right)]$$

Supposing *R* unlinked autosomal loci were used, the probability that two unrelated persons sharing at least one allele at all loci by chance could be taken as RPFM_*R*_(*θ*).

$${\text{RPFM}}_{\text{R}}\,\left(\theta \right)=\prod_{r=1}^{R}\left(1-Q_{r}\,\left(\theta \right)\right)$$

For the high mutation rate of STR, if inconsistency on a single locus was allowed, the matching probability between two unrelated persons could be RPFM’_*R*_(*θ*) (Yu and Fung, 2018).

$${\text{RPFM}}_{\text{R}}^{{}^{\prime }}\,\left(\theta \right)=\prod_{r=1}^{R}\left(1-Q_{r}\,\left(\theta \right)\right)+\sum_{s=1}^{R}Q\,s\,\left(\theta \right)\,\prod_{\begin{array}{c} r=1\\ r\neq s \end{array}}^{R}\left(1-Q_{r}\,\left(\theta \right)\right)$$

### Parentage Assignment

The paternity index (PI) was calculated as the ratio of likelihood values of two hypotheses (H0: test man is the biological father of the child; H1: test man is unrelated) based on the local allele frequency (Wu et al., 2016). The CPI is a product of PI in *R* unlinked loci. According to the Chinese national technical specification for parentage testing (Parentage Testing, 2016), inclusion of the parenthood is noted when the CPI is greater than 10^4^, and exclusion is noted when CPI is less than 10^–4^. Genetic inconsistencies at three or more STR loci are also required before exclusion.

### Genotyping With SNP Panel

Based on the panel CHP5457 previously developed in our laboratory (Chang et al., 2019), an optimized version CHP4830 consisting of 4,830 autosomal IISNPs was constructed and employed in this study. All SNPs in this panel met the following requirements: (1) distance from protein-coding genic region greater than 100 kb; (2) under HWE (*p* > 0.05) in Chinese Han; and (3) any two SNPs located on the same chromosome were in linkage equilibrium (*r*^2^ < 0.05). In the Chinese Han population, 98% SNPs in CHP4830 have a validated MAF value greater than 0.30.

The CHP4830 panel was used to validate all ambiguous cases according to the following approach. First, genome DNA was extracted using TIANamp Micro DNA Kit (TIANGEN, China) according to the manufacturer’s protocol. One hundred nanograms of DNA was fragmented to 200–500 bp by the endonuclease. When going through end blunt, an “A” tail and adaptor was ligated to DNA fragments, and then DNA libraries were prepared. After that, target SNP regions were captured with probes and sequenced on the BGISEQ-500 platform (BGI, China) with a PE50 strategy.

The raw sequencing reads were filtered out if they met one of these criteria: (1) N-content more than 1%; (2) reads overlapping >10 bp with the adapter sequence; (3) fraction of Q20 base less than 90%, and (4) duplicates derived from amplification. The obtained clean reads were aligned to human genome hg19^1^ using the Burrows-Wheeler Aligner aln algorithm (v0.7.12). Finally, the Genome Analysis Toolkit UnifiedGenotyper was utilized to perform SNP calling for loci with coverage >30-fold.

## Results

### False Inclusion Between Close Relatives

To investigate the incidence of ambiguous or false inclusion in relatives, a total of 75 duos of the child and first-degree relatives (parent, offspring, and full sibling) of the true parent were recruited. All samples were genotyped with HumDNA Typing-YanHuang (FGI, Shenzhen, China). For each pair, the Mendelian incompatibilities were counted, and the CPI was calculated. Our results showed that 2 of 29 grandparent–grandchildren pairs showed one mismatch, and 1 of 20 uncle–niece pairs showed two mismatches. For 26 full sibling pairs, 1 pair matched in all loci, 3 pairs showed only one mismatch, and 7 pairs showed 2 mismatches. In total, 8.0% (6 of 75) close relative pairs showed no more than one mismatch (Table 1), in which the close relative would be false inclusive as parent. If taking pairs with two or fewer mismatches as ambiguous results, the incidence of ambiguous paternity between close relatives is 18.67% (14 of 75). Details of mismatches are listed in Supplementary Table S1.

**TABLE 1:** Mendelian incompatibilities in pair-wised close relatives using STRs.

**Relative**	**No. of total pairs**	**No. of pairs with *N* incompatible STR loci**			
		**0-locus**	**1-locus**	**2-loci**	**3-loci**
Full sibling	26	1	3	7	6
Grandparent	29	0	2	0	2
Uncle/aunt	20	0	0	1	0
Parent-offspring	43	39	4	0	0
					

**FIGURE 1:**
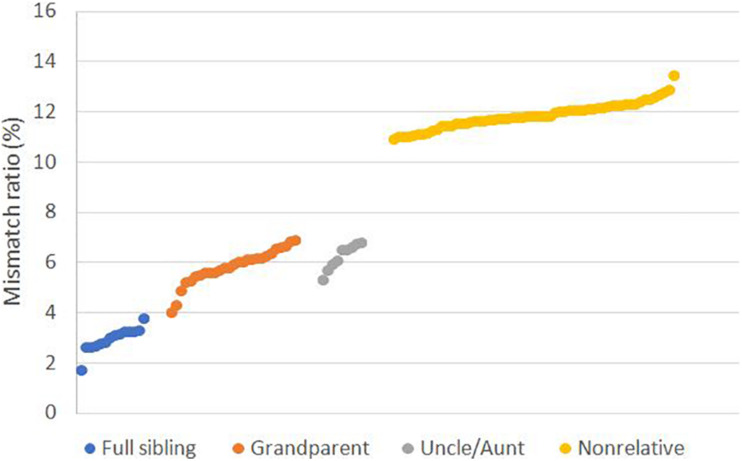
Mismatch ratio for relative and non-relative pairs based on SNP data. Mismatch ratio means the fraction of genetic incompatible SNPs in total detected loci. Tested relative types including full sibling (*n* = 14), grandparent-grandchild (*n* = 27), uncle/aunt-niece/nephew (*n* = 9), and non-relative (*n* = 60).

To evaluate the performance of SNPs in cases involving close relatives, we genotyped the samples with CHP4830. We calculated the mismatch ratio, the ratio of the number of mismatched loci to the total number of detected SNPs, and found the mismatch ratio 1.78–3.84% for full sibling was significantly lower than 4.07–6.95% for grandparents and 5.36–6.84% for uncle/aunt relationship, all of which were lower than 10.93–13.49% for non-relatives (Figure 1 and Supplementary Table S2).

To better understand the lower mismatch ratio, we calculated the theoretical possibility of not sharing an allele in a full sibling pair. If either father or mother or both are homozygous at one locus, full siblings certainly share alleles. As shown in Table 2, assuming under HWE and taking bi-allele SNP locus as example, the possibility that both parents are heterozygous is *4a^2^b^2^*. When both parents have heterozygous SNPs, the probability that a full sibling pair does not share an allele at this locus is 1/8. Thus, for a SNP to be tested, the possibility of not sharing an allele in a full sibling pair is *1/2a^2^b^2^* (*4a^2^b^2^*^∗^1/8). Taking all SNPs together, the theoretical value of mismatch ratio between full sibling pair is also *1/2a^2^b^2^* because these loci are unlinked. As nearly all SNPs in CHP4830 have a validated MAF range in 0.3 to 0.5 (Chang et al., 2019), the mismatch ratio between full sibling pair is between 2.21% and 3.13% theoretically (Table 2).

**TABLE 2:** Theoretical possibility of not sharing allele in full sibling pair using bi-allele SNPs.

		**Genotype possibility**			
**Allele frequency^*a*^**		**F (AB)&& M(AB)**	**C1(AA) && C2(BB)**	**C1(BB) && C2(AA)**	**Possibility of not sharing allele in full sibling pair**
**a**	**b (MAF)**	**4a^2^b^2^**	1/16*4a^2^b^2^	1/16*4a^2^b^2^	**1/2a^2^b^2^**
0.5	0.5	/	/	/	3.13%
0.6	0.4	/	/	/	2.88%
0.7	0.3	/	/	/	2.21%

*^a^a and b stand for the frequency of alleles A and B, respectively.*

### Random Match Between Non-relatives

To assess the probability of random match in unrelated individuals, data of 20 autosomal STRs with STRtyper-21G (HEALTH Gene Technologies, China) from 3,955 individuals were collected after approval. Ignoring non-DNA information, such as age, gender, and district, STR data of each person were paired with all the others. After excluding the registered relatives, 7,818,969 comparisons were obtained. For each pair, the number of Mendelian incompatibilities was counted.

Eventually, there were 33 perfectly matched pairs, in which two unrelated participants share an allele at all loci totally by chance, and the observed probability of RPFM is 4.22 × 10^–6^. Note that two samples matched twice with non-relatives. Subsequently, we used CHP4830 to genotype these random matched samples, then calculated the mismatch ratio in each pair. The result showed that the fraction of mismatch loci to total SNPs was between 10.04% and 13.29% (Supplementary Tables S3, S4), which was equivalent to the ratio 10.93–13.49% in unrelated pairs in Figure 1. It suggested that these 33 pairs were indeed random false matches between non-relatives.

According to the calculation method from Ayres (2002) and Yu and Fung (2018), when using kit STRtyper-21G, the theoretical RPFM value is 3.64 × 10^–6^ under HWE (*θ* = 0) and 1.08 × 10^–5^ in the substructure population (*θ* = 0.02), which is consistent with our observed value 4.22 × 10^–6^ (Table 3). Considering the high mutation rate of STR, if single locus inconsistency was allowed, 499 additional random match pairs could be found, and the observed RPFM value would increase to 6.80 × 10^–5^ (Table 3).

**TABLE 3:** Probability of random Mendelian matching in non-relative duos with 20 STRs in STRtyper-21G kit under HWE and population substructure.

**Random matching degree**	**Theoretical value**		**Observed value**
	***θ* = 0**	***θ* = 0.02**	
No mismatch	3.64E–06	1.08E–05	4.22E–06
One mismatch allowed	7.09E–05	1.86E–04	6.80E–05

### Case Rectifying

For ambiguous cases with insufficient CPI accompanied with separate genetic incompatibilities, most can be correctly determined by combining different STR kits. However, here we collected three real cases in which the paternity could easily be misjudged based on STRs. By analyzing with CHP4830, a powerful statistical evidence favoring one of the alternative hypotheses can be given for each case (Table 4).

**TABLE 4:** Data summary of three ambiguous cases in duos.

**Case no.**	**STR**				**SNP (CHP4830)**		
	**Mismatches/total loci**	**CPI**	**Paternity^*a*^**	**Kit**	**Mismatches/total loci**	**CPI**	**Paternity**
I	0/19	1.65E+05	Y	EX20	297/4,350	5.87E–1420	N
	3/17	3.42E–06	N	HumDNA			
II	2/20	1.28E–04	A	21G	273/4,807	4.43E–1163	N
	0/22	6.62E+06	Y	23sp			
III	2/17	1.52E–03	A	HumDNA	48/4,150^*b*^	2.38E+309^*c*^	Y

*^a^Y means inclusion paternity, N means excluded, A means ambiguous.*

*^*b*^All 48 mismatches were located on chromosome 13.*

*^*c*^CPI computed after excluding all SNPs on chromosome 13.*

In case I, opposite conclusions were drawn by two different commercial STR kits. For kit 1 (EX20, AGCU), child and alleged father shared alleles in all 19 loci, with a CPI of 1.65E+05 (Supplementary Table S5). However, for kit 2 (HumDNA Typing, FGI), three genetic incompatibilities (D18S1364, D11S2368, and D13S3253) were observed, and the CPI was 3.42E–06 (Supplementary Table S6). By using CHP4830, 297 genetically inconsistent loci were found in 4,350 SNPs. With a conservative mutation rate of 10^–6^ for SNPs, the CPI was 5.87E–1420; thus, the alleged father was excluded as being the true one (Table 4 and Supplementary Table S7).

In case II, for kit 1 (STRtyper-21G, HEALTH Gene Technologies), two genetic inconsistencies (PentaE, TPOX) between child and alleged mother were found in 20 autosomal STRs, and the CPI was 1.28E–04; thus, no clear conclusion could be drawn (Supplementary Table S8). For kit 2 (Microreader 23sp ID System), no incompatibility loci were found in a total of 22 STRs, with a CPI value 6.62E+06 larger than 10,000 (Supplementary Table S9). For SNP data, 273 genetically inconsistent loci were found in 4,807 SNPs, the CPI was 4.43E–1163, and the alleged parent was excluded (Table 4 and Supplementary Table S10).

In case III, two genetic inconsistencies (D13S317 and D13S325) were found with a CPI 1.52E–03 by kit HumDNA Typing (FGI). As the child was homozygous at both loci, we inferred that the incompatible loci may result from allele dropout (Supplementary Table S11). Of 4,150 detected SNPs, 48 genetic inconsistencies were found between the child and the alleged parent. We noticed that all 48 inconsistent SNPs were located on chromosome 13 q arm (coordinate of chr13q on hg19: 17,900,000–115,169,878), and the genotypes of the child on these SNPs were all homozygous; thus, we concluded that these mismatches may result from a partial chromosome deletion in the child. After removing all SNPs on chromosome 13, no genetic inconsistencies were found, and the CPI was 2.38E+309, so the paternity should be included (Table 4 and Supplementary Table S12).

## Discussion

In undetermined cases based on STRs, the most highly considered possibility is that the putative parent is actually a close relative to the real parent, especially in parentage testing for immigration purposes (Wenk and Shao, 2014). When considering a close relative as a putative parent, the probability of ambiguous or false paternity was as high as 18.67%. As they are more likely to share an allele, the incidence of false inclusion between full siblings is higher than grandparents and uncles/aunts. With the GlobalFiler^TM^ system, Ochiai predicted that the probability of matches with only 1 mismatch in 21 STRs for full siblings was 23.5% (Ochiai et al., 2016). Lee simulated families with 15 STRs in silico; the false inclusion rate was 19.0% when considering a sibling as the parent (Lee et al., 2013). These data suggested that the current STR systems are insufficient to distinguish a close relative from the true parent in deficiency cases, and supplementary investigations need to be performed.

Fifty to 60 SNPs are considered to be comparable to 15 STRs in discriminatory power (Borsting et al., 2008). Thus, many laboratories use approximately 50 SNPs as supplementary markers to solve ambiguous STR results (Borsting and Morling, 2011; Lindner et al., 2014). It was reported that older and younger siblings occasionally pretend to be parent and child to expedite immigration (Wenk and Shao, 2014). According to the observed and theoretical mismatch ratio in close relatives, extending results beyond the commonly used ∼50 SNPs should be interpreted with caution, because there is still a considerable probability of false inclusion when a possible relative is involved in parentage testing.

**FIGURE 2:**
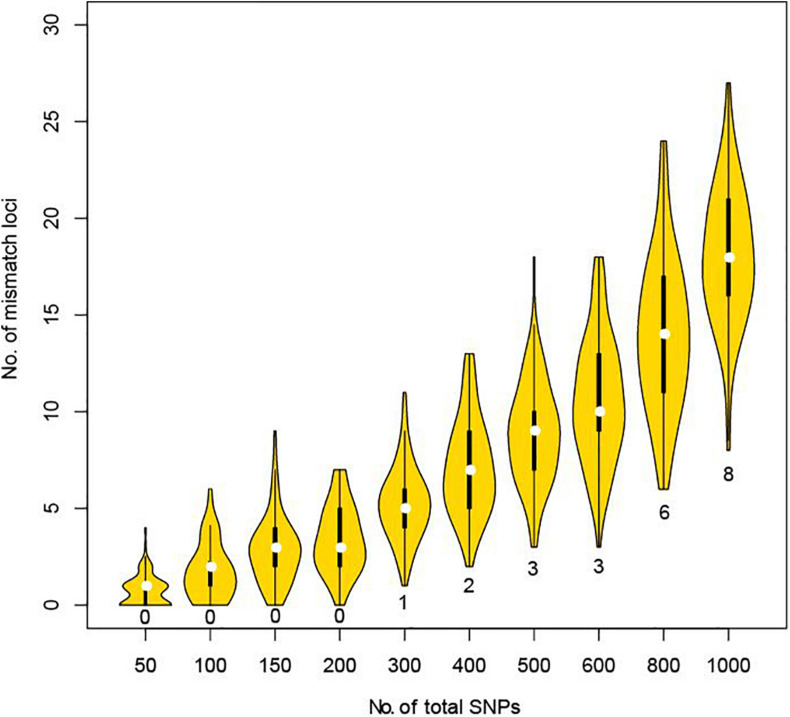
Mismatch loci number under different total detected SNPs in simulation between full sibling pair. For each violin, we randomly selected the corresponding number of SNPs and counted the mismatch loci number for 100 times. Minimum of mismatch loci were labeled for each violin.

Using the CHP4830 panel can greatly facilitate the distinction between a close relative and the true parent. But considering cost and efficiency, we further explored how many SNPs are sufficient to distinguish close relatives from true parents by simulation experiments. A greater proportion of alleles are shared between two full siblings than other pairs of relatives. Using genotyping data from full sibling pair with a low mismatch ratio (1.78%), we took 100 random samples of 50, 100, 150, 200, 300, 400, 500, 600, 800, or 1,000 SNPs and counted the number of mismatch loci. No mismatches were found when sampling up to 200 SNPs. When sampling 300–600 SNPs, a minimum of 1–3 mismatches were found. When continuing to extend the simulation, 6–24 mismatches were found when sampling up to 800 SNPs, and 8–27 mismatches found when sampling up to 1,000 SNPs (Figure 2). When using STRs, it is recommended that more than three loci are required before exclusion (Parentage Testing, 2016). Therefore, we recommend that, when lacking genetic background information or involving possible relatives, at least 800–1,000 unlinked SNPs distributed on all autosomes with a MAF larger than 0.3 should be used to address vague parental issues in deficiency cases.

Compared with ambiguous paternity in close relatives, random false match between non-relatives was relatively rare in routine casework. Thus, little attention was paid to the RPFM matter previously. Our observed RPFM ratio was 6.80 × 10^–5^ when one mismatch is allowed, which is comparable to the predicted match probability 8.59 × 10^–5^ between non-relatives by using 21 STRs in the GlobalFiler^TM^ system (Ochiai et al., 2016). Human trafficking remains a serious global social problem in modern society and is an important issue that needs to be solved. According to the statistics from the International Organization for Migration (IOM), approximately one in three victims is a child (Trafficking of Children). So taking anti-trafficking as an example, when searching 100 returned children in a database of one million persons, there will be around 6,800 random matches in 10^8^ searching times. To distinguish the true match from thousands of random matches, laborious work such as investigating the information of gender and age and collecting information of more family members is required. It is reasonable to infer that, if more individuals are included in the database or there are more complex pedigrees subject to endogamous relations, the outcome might be more difficult with the currently used STR kits. Repeating the above simulations for RPFM pair, when 100 SNPs were detected, 4–17 mismatches could be found (Supplementary Figure S1). To effectively distinguish random match between non-relatives, at least 100 supplementary SNPs are recommended to combine with STR kit. Moreover, haplotypes of STR or SNP on the Y chromosome could also improve discrimination power between non-relative male pairs (Fu et al., 2020).

In forensic casework, apart from occasional reported chimerism-induced false exclusions of paternity (Sheets et al., 2018), using more STR loci to increase the confidence is the most commonly used strategy when facing an ambiguous STR result. However, three cases rectified with SNP panel in this study should arouse vigilance. First, more STRs may give opposite or even misjudged conclusion. In addition, abnormal chromosome structure also produced confusing STR results. A genome-wide high density SNP panel could provide key supplementary markers to traditional STRs because they can give clearer conclusions in these situations.

## Data Availability Statement

The datasets presented in this study can be found in online repositories. The names of the repository/repositories and accession number(s) can be found in the article/Supplementary Material.

## Ethics Statement

The studies involving human participants were reviewed and approved by the Institutional Review Board Administration in Xi’an Jiaotong University (No. 2018-408). The patients/participants provided their written informed consent to participate in this study. Written informed consent was obtained from the individual(s) for the publication of any potentially identifiable images or data included in this article.

## Author Contributions

LC conceived and designed the experiments and wrote the manuscript. XM and SW performed the experiments. HY and LC were involved in the data analysis. BZ and SL revised the manuscript. All authors had read and approved the final manuscript.

## Conflict of Interest

The authors declare that the research was conducted in the absence of any commercial or financial relationships that could be construed as a potential conflict of interest.

## Footnotes

1 http://hgdownload.soe.ucsc.edu/goldenPath/hg19/bigZips/

## Supplementary Material

Supplementary Figure S1 Mismatch loci number under different total detected SNPs in simulation between RPFM pair. For each violin, we randomly selected the corresponding number of SNPs and counted the mismatch loci number, for 100 times. Minimum of mismatch loci were labeled for each violin.
