# Supplementary figures and images for: Evaluation of a Custom SNP Panel for Identifying and Rectifying of Misjudged Paternity in Deficiency Cases
Source: Front Genet. 2021 Feb 22;12:602429. doi: 10.3389/fgene.2021.602429 (PMC7937934; doi:10.3389/fgene.2021.602429)

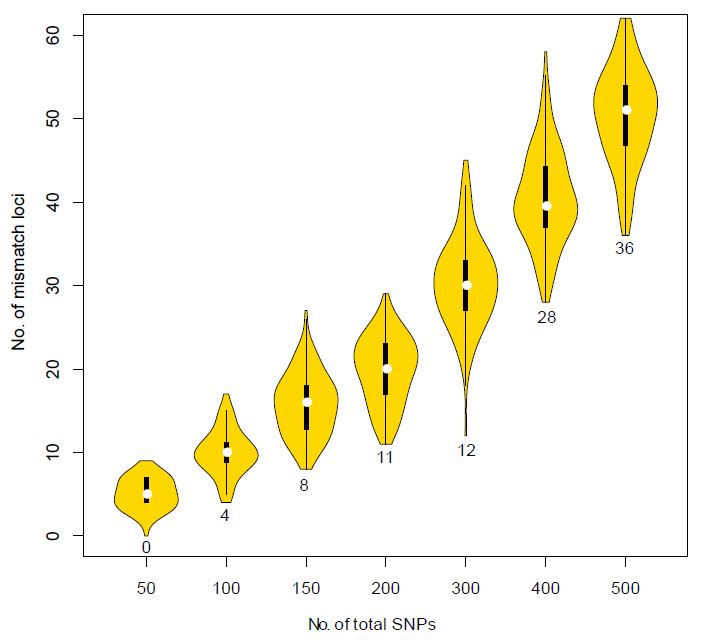

Supplement: Supplementary Figure S1 — Mismatch loci number under different total detected SNPs in simulation between RPFM pair. For each violin, we randomly selected the corresponding number of SNPs and counted the mismatch loci number, for 100 times. Minimum of mismatch loci were labeled for each violin. [file Image_1.JPEG]
